# Plants, Pills, and the Brain: Exploring Phytochemicals and Neurological Medicines

**DOI:** 10.26502/ijpaes.4490180

**Published:** 2025-08-04

**Authors:** Arnav Aggarwal, Resmi Rajalekshmi, Anshu Aggarwal, Devendra K. Agrawal

**Affiliations:** 1 Loveless Academic Magnet Program, Montgomery, AL, USA; 2 Department of Translational Research, College of Osteopathic Medicine of the Pacific, Western University of Health Sciences, Pomona, CA 91766, USA; 3 Department of Biology and Environmental Sciences, College of Science, Auburn University at Montgomery, Montgomery, AL 36117, USA

**Keywords:** Alkaloids, Alzheimer’s disease, Epilepsy, Flavonoids, Neurodegenerative disease, Neurological disorders, Parkinson’s disease, Phytochemicals, Spinal cord injuries, Terpenoids, Traumatic brain injury

## Abstract

Neurological disorders, such as Alzheimer’s disease, Parkinson’s disease, epilepsy, spinal cord injuries, and traumatic brain injuries, represent substantial global health challenges due to their chronic and often progressive nature. While allopathic medicine offers a range of pharmacological interventions aimed at managing symptoms and mitigating disease progression, it is accompanied by limitations, including adverse side effects, the development of drug resistance, and incomplete efficacy. In parallel, phytochemicals—bioactive compounds derived from plants—are receiving increased attention for their potential neuroprotective, antioxidant, and anti-inflammatory properties. This review will explore the therapeutic landscape of neurological diseases by providing a comprehensive overview of prevalent conditions and the current allopathic treatments available. Furthermore, this review will investigate specific phytochemicals, including flavonoids, alkaloids, and terpenoids, that exhibit promise in modulating various disease pathways. Emphasis will be placed on the interactions between plant-derived compounds and prescription medications, highlighting both potential synergistic effects and possible adverse interactions. A thorough understanding of these interactions is essential for the development of integrative treatment approaches that enhance therapeutic efficacy while minimizing harm. By bridging traditional herbal medicine with contemporary pharmacotherapy, this review aims to promote a more holistic perspective on the management of neurological diseases, while also encouraging further research into safe and effective combinatory therapies.

## 1. Introduction

Neurological diseases, as defined by the World Health Organization, are central and peripheral nervous disorders. The central nervous system includes the brain and spinal cord, while the peripheral nervous system is a network of nerves that extends throughout the head, neck, and body. These diseases can be grouped into several categories, such as neurodegenerative disorders (e.g., Alzheimer's and Parkinson's), neuromuscular conditions, brain disorders (like epilepsy), and spinal cord conditions. They often lead to progressive damage that affects memory, movement, and overall daily activity.

While allopathic medicine, conventional Western medical treatment, primarily uses pharmaceutical drugs and procedures to manage these conditions, it may not have long-term solutions or help address the underlying causes. In contrast, phytochemicals, naturally occurring compounds in plants known for their potential health benefits, have become of interest. These substances are found in fruits, vegetables, herbs, and other plant sources, which may influence brain function and protect nerve cells. It is essential to study how phytochemicals interact with modern allopathic treatment, as it may reveal complementary effects, reduce side effects, and help create more effective treatments for neurological diseases.

## 2. Overview of Neurological Disease

**Figure 1:**
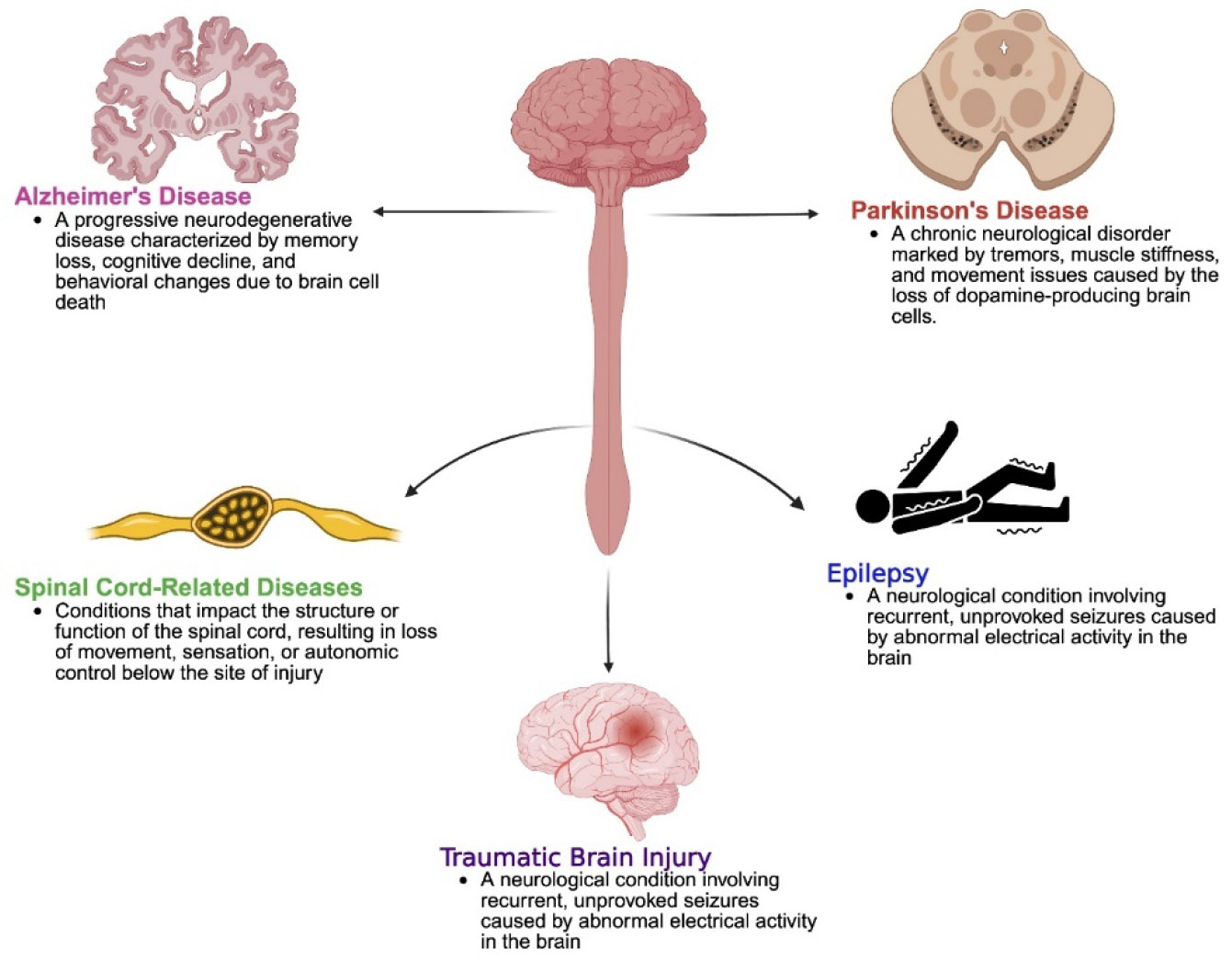
Classification and overview of various neurological diseases.

Neurological disorders encompass a wide range of conditions that affect the brain, spinal cord, and nerves. These diseases can be progressive, episodic, or chronic, and they often result in a combination of cognitive, motor, behavioral, and sensory impairments. Among the most prevalent and impactful neurological conditions are Alzheimer’s disease, Parkinson’s disease, epilepsy, and traumatic brain injury (Figure 1). Each of these disorders exhibits distinct etiologies and pathologies, yet many share overlapping features of neuronal damage, neuroinflammation, and neurodegeneration.

### 2.1 Alzheimer’s Disease

Alzheimer’s disease (AD) is the most prevalent form of dementia, accounting for an estimated 60–80% of cases globally. It is a progressive neurodegenerative condition characterized by memory loss, disorientation, and impaired reasoning. The hallmark pathological features include extracellular amyloid-beta (Aβ) plaque accumulation and intracellular neurofibrillary tangles formed from hyperphosphorylated tau protein. These abnormalities lead to synaptic dysfunction, neuronal death, and cerebral atrophy, particularly in the hippocampus and cortex [1].

Cognitive deficits are often preceded by mild cognitive impairment (MCI), which may progress over years. Neuroinflammation and oxidative stress are central to the pathogenesis, with microglial activation playing a dual role in both clearing amyloid and propagating damage. Genetic factors, such as mutations in APP, PSEN1/2, and the APOE ε4 allele, increase disease risk [2,3].

### 2.2 Parkinson’s Disease

Parkinson’s disease (PD) is the second most common neurodegenerative disorder after Alzheimer’s, primarily affecting motor control. It is marked by the degeneration of dopaminergic neurons in the substantia nigra pars compacta, leading to dopamine deficiency in the striatum. Neuropathologically, PD is associated with Lewy bodies—cytoplasmic inclusions composed mainly of misfolded alpha-synuclein protein [4,5].

Clinical symptoms begin insidiously, with tremor at rest, bradykinesia, rigidity, and postural instability. Non-motor manifestations—including depression, anosmia, sleep disturbances, and cognitive impairment—often precede motor symptoms and can dominate the disease burden [6,7].

Although the etiology is multifactorial, genetic mutations in SNCA, LRRK2, and PINK1, as well as environmental exposures to toxins like pesticides, have been implicated. A growing body of evidence links gut microbiota and alpha-synuclein misfolding as early contributors to pathogenesis [8].

### 2.3 Epilepsy

Epilepsy is a neurological disorder marked by the tendency for recurrent, unprovoked seizures due to abnormal, excessive neuronal activity in the brain. It is a heterogeneous condition with multiple subtypes classified based on seizure type (e.g., focal, generalized), etiology, and electroclinical features [9].

Seizures can range from subtle lapses in consciousness (absence seizures) to dramatic convulsions involving the entire body (tonic-clonic seizures) [10]. Depending on the brain region involved, seizures may present with sensory disturbances, automatisms, motor abnormalities, or behavioral changes. The underlying causes of epilepsy include genetic mutations, brain injury, infections, developmental disorders, and metabolic conditions. In many cases, the etiology remains idiopathic [11].

### 2.4 Traumatic Brain Injury

Traumatic brain injury (TBI) results from an external mechanical force that causes brain dysfunction. TBIs can range from mild concussions to severe brain damage and are categorized based on the mechanism (blunt or penetrating), severity, and the affected brain region [12].

The pathophysiology of TBI involves both primary injury, which occurs at the moment of impact, and secondary injury, which evolves over time due to processes like cerebral edema, ischemia, excitotoxicity, and neuroinflammation [13,14]. TBI can cause diffuse axonal injury, contusions, hemorrhages, and disruption of the blood-brain barrier.

Clinical manifestations vary widely and may include loss of consciousness, confusion, amnesia, headache, dizziness, behavioral changes, and cognitive impairment. Severe TBIs are associated with long-term neurological deficits and an increased risk of developing neurodegenerative conditions like Alzheimer’s disease and Parkinson’s disease [15,16].

Additionally, repetitive mild TBIs—such as those sustained in contact sports—are linked to chronic traumatic encephalopathy (CTE), a degenerative brain disease marked by behavioral abnormalities, mood disturbances, and cognitive decline. Studies also show that TBI may precipitate late-onset psychiatric conditions and accelerate neurodegenerative processes [17].

## 3. Phytochemicals

Phytochemicals, also termed phytonutrients, encompass a structurally diverse group of secondary metabolites synthesized by plants via complex biosynthetic pathways. Unlike essential micronutrients, these compounds are not required for human survival but exhibit significant bioactive properties, including antioxidant, anti-inflammatory, antimicrobial, anticancer, and neuroprotective activities. Their diversity, spanning polyphenols, flavonoids, carotenoids, alkaloids, terpenoids, glucosinolates, and saponins, reflects extensive structural and functional heterogeneity [18].

Polyphenols form one of the most abundant and researched categories, including flavonoids, phenolic acids, stilbenes, and lignans. Flavonoids—such as flavonols (quercetin, kaempferol), flavanols (catechins), flavones, isoflavones, and anthocyanins—are particularly notable for their C6–C3–C6 structural motif that influences antioxidant behavior and interaction with cellular signaling pathways [19]. Anthocyanins, common in berries, red cabbage, and purple corn, contribute visual pigmentation and have demonstrated cardioprotective and anti-inflammatory properties [20].

Carotenoids, including β-carotene, lutein, and lycopene, are isoprenoid pigments that function in photoprotection and oxidative stress modulation. They are precursors to vitamin A and exhibit protective effects against chronic eye and cardiovascular diseases. Terpenes and terpenoids, structurally derived from five-carbon isoprene units, include compounds such as artemisinin, menthol, and saponins—exhibiting antimalarial, antimicrobial, and lipid-lowering activities [21].

Alkaloids, nitrogen-containing molecules like morphine, caffeine, and berberine, occur predominantly in medicinal plants and are well-documented for pharmacological activities in traditional and modern medicine [22]. Glucosinolates, characteristic of Brassicaceae vegetables, degrade into biologically active isothiocyanates—potent agents for chemoprevention through epigenetic modulation and detoxification enzyme activation [23].

Despite compelling *in vitro* and *in vivo* preclinical data, the translational potential of phytochemicals is frequently constrained by issues of solubility, absorption, metabolism, and bioavailability. Strategies to overcome these limitations include nanoformulation, prodrugs, complexation with cyclodextrins, and co-administration with absorption enhancers. Phytochemicals have shown efficacy in the chemoprevention and adjunct treatment of various cancers, neurodegenerative diseases, and metabolic disorders.

## 4. Common Allopathic Treatments for Neurological Diseases

Allopathic medicine combines neurological diseases with pharmaceutical drugs and specialized therapies. These treatments are for managing symptoms, slowing disease progression, and improving quality of life.

### 4.1 Alzheimer's Disease

**Figure 2:**
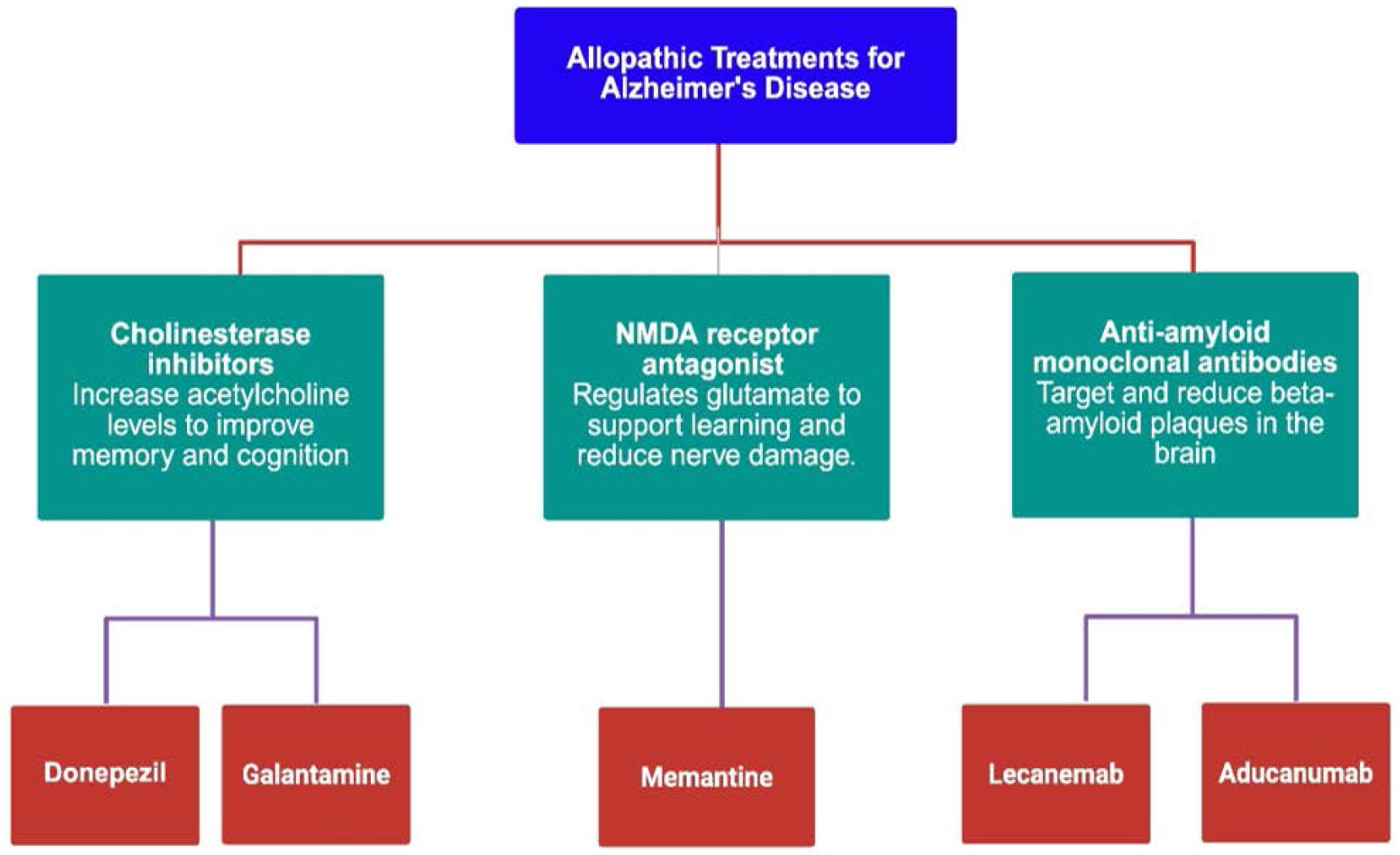
Categories of allopathic drugs used in the management of Alzheimer’s Disease.

Alzheimer’s disease (AD) is managed using a spectrum of pharmacological agents aimed at alleviating symptoms and modifying disease progression. The various classes of allopathic drugs used are illustrated in Figure 2.

Cholinesterase inhibitors (ChEIs) such as donepezil and galantamine are commonly prescribed to enhance cholinergic neurotransmission by inhibiting acetylcholinesterase (AChE), thereby increasing acetylcholine levels in synapses. Galantamine, notably derived from *Amaryllidaceae* plants, also exhibits nicotinic receptor allosteric modulation and contains bioactive alkaloids that may contribute to its efficacy [24,25].

The NMDA receptor antagonist memantine is another cornerstone treatment, particularly for moderate-to-severe AD. Memantine modulates glutamatergic activity by antagonizing NMDA receptors, thereby preventing excitotoxic neuronal damage caused by excessive glutamate—a hallmark in AD pathology. It is well-tolerated and often combined with donepezil to target both cholinergic and glutamatergic dysfunctions [26,27].

In terms of disease-modifying therapies (DMTs), the recent introduction of anti-amyloid monoclonal antibodies represents a significant advancement. Aducanumab, a monoclonal antibody targeting aggregated amyloid-β (Aβ42), preferentially binds to fibrillar deposits and was the first FDA-approved DMT in June 2021 for early-stage AD patients with confirmed amyloid pathology. Despite controversies regarding its clinical benefit and approval process, aducanumab marked a paradigm shift in AD management by targeting pathophysiological mechanisms rather than symptoms alone [28,29].

Lecanemab, another FDA-approved monoclonal antibody, selectively binds to soluble Aβ protofibrils. It was derived from the mouse mAb158 and received accelerated FDA approval in January 2023, followed by full approval in July 2023. Lecanemab is indicated for patients with mild cognitive impairment or mild AD dementia and confirmed amyloid pathology. Its biweekly intravenous regimen and well-defined targeting profile make it a promising DMT option, although concerns remain about amyloid-related imaging abnormalities (ARIA) and long-term safety [30].

Overall, while agents like donepezil, galantamine, and memantine offer symptomatic relief, monoclonal antibodies such as aducanumab and lecanemab represent an emerging strategy focused on modifying disease progression. However, clinical application necessitates early diagnosis, amyloid positivity confirmation, and monitoring for adverse effects—highlighting the evolving landscape of Alzheimer’s disease therapeutics [31,32].

### 4.2 Parkinson's Disease

**Figure 3:**
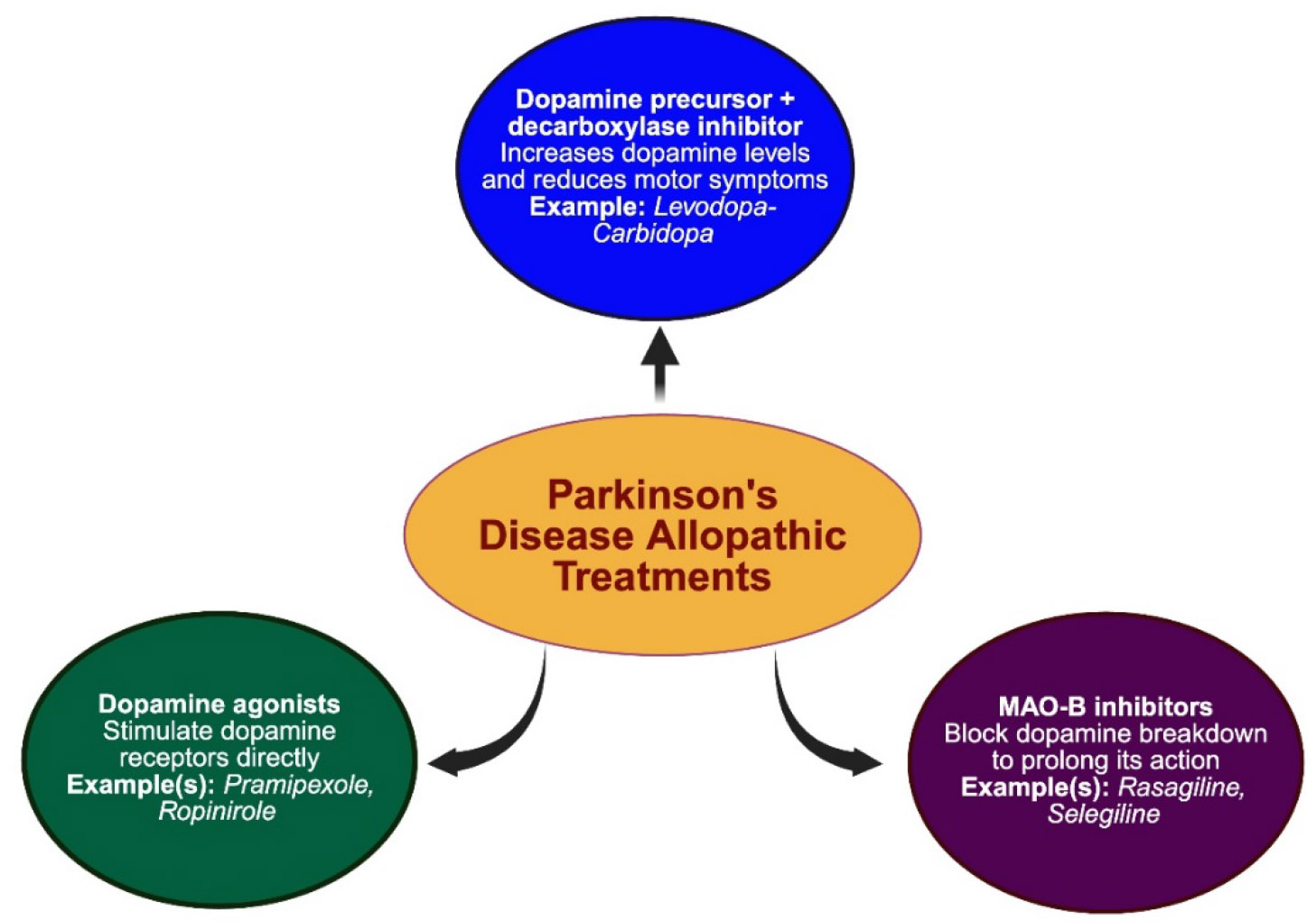
Categories of allopathic drugs used in the management of Parkinson’s disease. MAO, monoamine oxidase.

Parkinson’s disease (PD) is a neurodegenerative disorder characterized by the progressive loss of dopaminergic neurons in the substantia nigra. The various classes of allopathic drugs used for PD are illustrated in Figure 3.

The first-line treatment remains levodopa, often administered with carbidopa to inhibit peripheral metabolism and enhance central nervous system delivery. Levodopa acts as a dopamine precursor and significantly improves motor symptoms such as bradykinesia, rigidity, and tremor. Although generally well tolerated, levodopa-carbidopa therapy may induce long-term side effects including dyskinesias, hallucinations, and gastrointestinal disturbances [33].

In early or fluctuating stages of PD, dopamine agonists such as pramipexole and ropinirole are often employed. These agents selectively activate D2-like dopamine receptors and can delay the initiation of levodopa therapy. Pramipexole and ropinirole have demonstrated significant efficacy in reducing motor symptoms and improving quality of life, although they are associated with adverse effects such as impulse control disorders, orthostatic hypotension, and sleep disturbances [34,35].

Another key pharmacological approach includes monoamine oxidase-B (MAO-B) inhibitors, specifically rasagiline and selegiline. These agents inhibit the enzymatic degradation of dopamine, thereby prolonging its synaptic availability. Rasagiline, in particular, has been shown to exert greater potency than selegiline and has demonstrated disease-modifying potential in early PD when used at 1 mg/day [36].

Overall, pharmacologic management of PD requires a tailored approach that evolves with disease progression. Combination therapies, such as dopamine agonists with levodopa or adjunctive MAO-B inhibitors, are commonly employed to mitigate motor complications and enhance therapeutic efficacy.

### 4.3 Epilepsy

**Figure 4:**
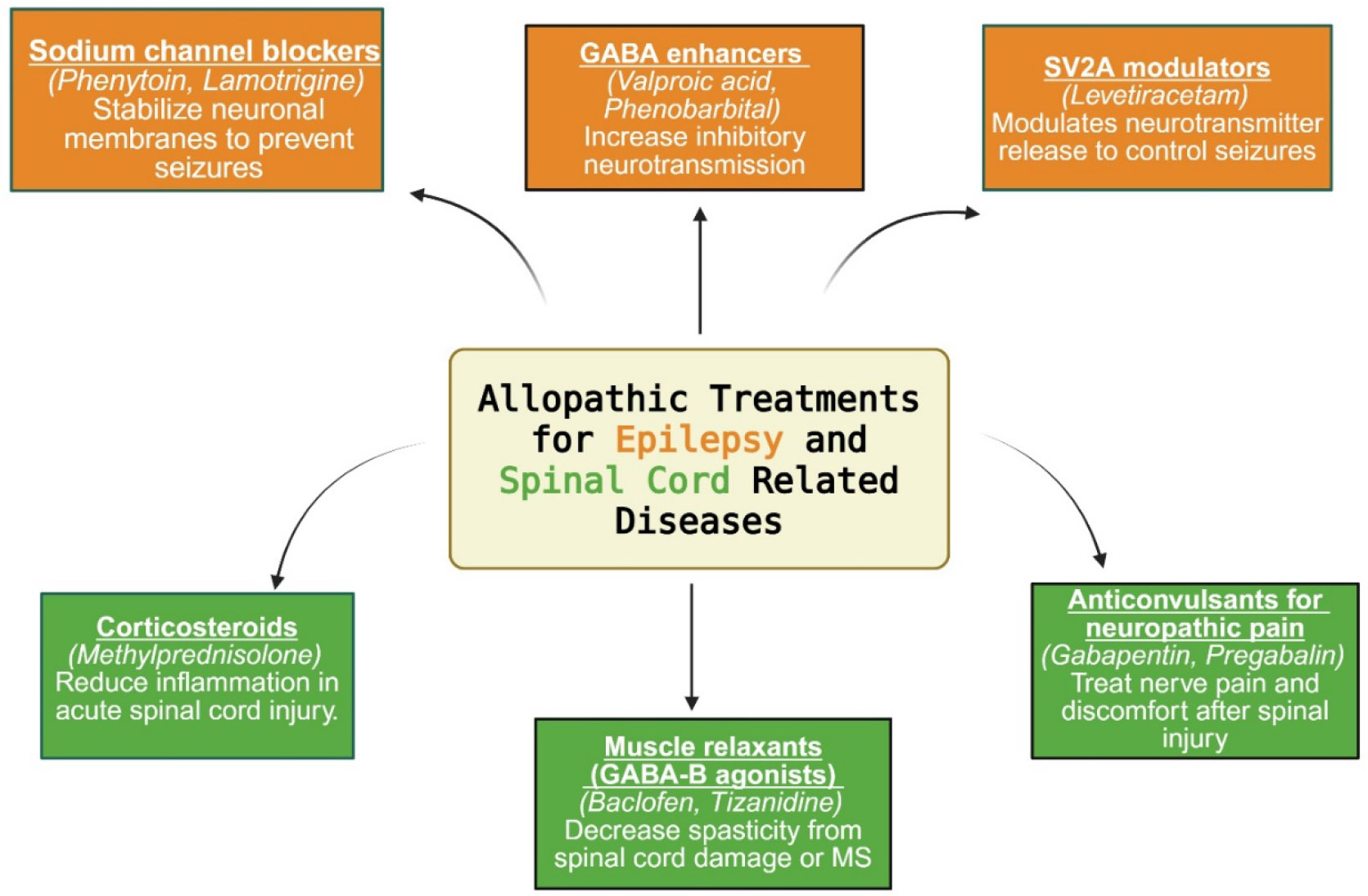
Categories of allopathic drugs used in the management of Epilepsy and Spinal cord injury. GABA, gamma amino butyric acid; MS, multiple sclerosis; SCI, spinal cord injury; SV2A, synaptic vesicle glycoprotein 2A.

The management of epilepsy primarily involves long-term administration of antiepileptic drugs (AEDs) aimed at stabilizing neuronal excitability and preventing seizure recurrence. The various classes of allopathic drugs used for epilepsy are illustrated in Figure 4.

A major class of AEDs—sodium channel blockers—includes phenytoin and lamotrigine, which stabilize neuronal membranes by promoting the fast inactivation of voltage-gated sodium channels. Newer agents, such as lacosamide and eslicarbazepine acetate, act on the slow inactivation phase of sodium channels, potentially improving tolerability and reducing cognitive side effects at therapeutic doses [37,38].

Older drugs like phenytoin and carbamazepine remain effective but are often limited by enzyme induction and complex pharmacokinetics, prompting a shift toward newer agents with favorable safety profiles [39].

Modulstors of gamma amino butyric acid (GABA), including valproic acid and phenobarbital, exert their antiepileptic effect by enhancing GABAergic inhibition. These compounds increase chloride influx into neurons via GABA-A receptor activation, producing hyperpolarization and reducing neuronal excitability. Additional agents such as tiagabine (a GABA reuptake inhibitor) and vigabatrin (an irreversible inhibitor of GABA transaminase) offer alternative means to elevate synaptic GABA levels, contributing to seizure control—particularly in refractory or infantile epilepsy settings [40,41].

Another mechanistically distinct class includes modulators of synaptic vesicle glycoprotein 2A (SV2A), typified by levetiracetam, which bind to the SV2A. This membrane protein regulates calcium-dependent neurotransmitter release, especially of GABA and glutamate [42]. SV2A dysfunction has been implicated in epileptogenesis and developmental epileptic encephalopathies. Levetiracetam’s precise binding to SV2A facilitates broad-spectrum seizure control with minimal drug-drug interactions, making it a preferred option for both monotherapy and adjunctive use [43,44].

### 4.4 Spinal Cord-Related Diseases

Treatment of spinal cord-related disorders primarily targets inflammation, spasticity, and neuropathic pain to improve function and quality of life. The various classes of allopathic drugs used for spinal cord injury are illustrated in Figure 4. In acute spinal cord injury (SCI), high-dose methylprednisolone has historically been used to reduce inflammation and limit secondary neuronal damage, although its routine use is now controversial due to mixed outcomes and adverse effects [45]. Muscle relaxants, especially GABA-B receptor agonists like baclofen and tizanidine, are frequently used to manage spasticity associated with spinal cord injury or multiple sclerosis (MS). These drugs act centrally to inhibit excitatory neurotransmission at the spinal level, effectively reducing muscle tone and spasm frequency [46,47].

For neuropathic pain, particularly post-SCI, gabapentin and pregabalin—structural analogs of GABA—are frontline agents. They bind to the α2δ subunit of voltage-gated calcium channels, inhibiting excitatory neurotransmitter release. These agents are effective in managing pain and paresthesia, though they require dose titration to balance efficacy with side effects such as dizziness or sedation [48]. A distinct and debilitating complication, complex regional pain syndrome (CRPS), often arises after peripheral nerve trauma and is characterized by central sensitization, autonomic dysregulation, and neuroimmune inflammation. Dysregulated C-fiber signaling and increased adrenergic receptor expression contribute to symptoms such as allodynia and hyperalgesia [49]. Pharmacologic interventions in CRPS may include ketamine infusions (an NMDA receptor antagonist), bisphosphonates, low-dose naltrexone (an opioid receptor modulator with neuroinflammatory effects), and botulinum toxin A for regional pain relief. Ketamine has shown efficacy even in later stages of CRPS, while naltrexone's immunomodulatory properties are gaining recognition in chronic pain management [50,51]. Ultimately, optimal outcomes require multidisciplinary approaches that integrate pharmacotherapy with physical rehabilitation, psychosocial support, and interventional techniques to mitigate the long-term burden of spinal cord-related diseases.

### 4.5 Traumatic Brain Injury

**Figure 5:**
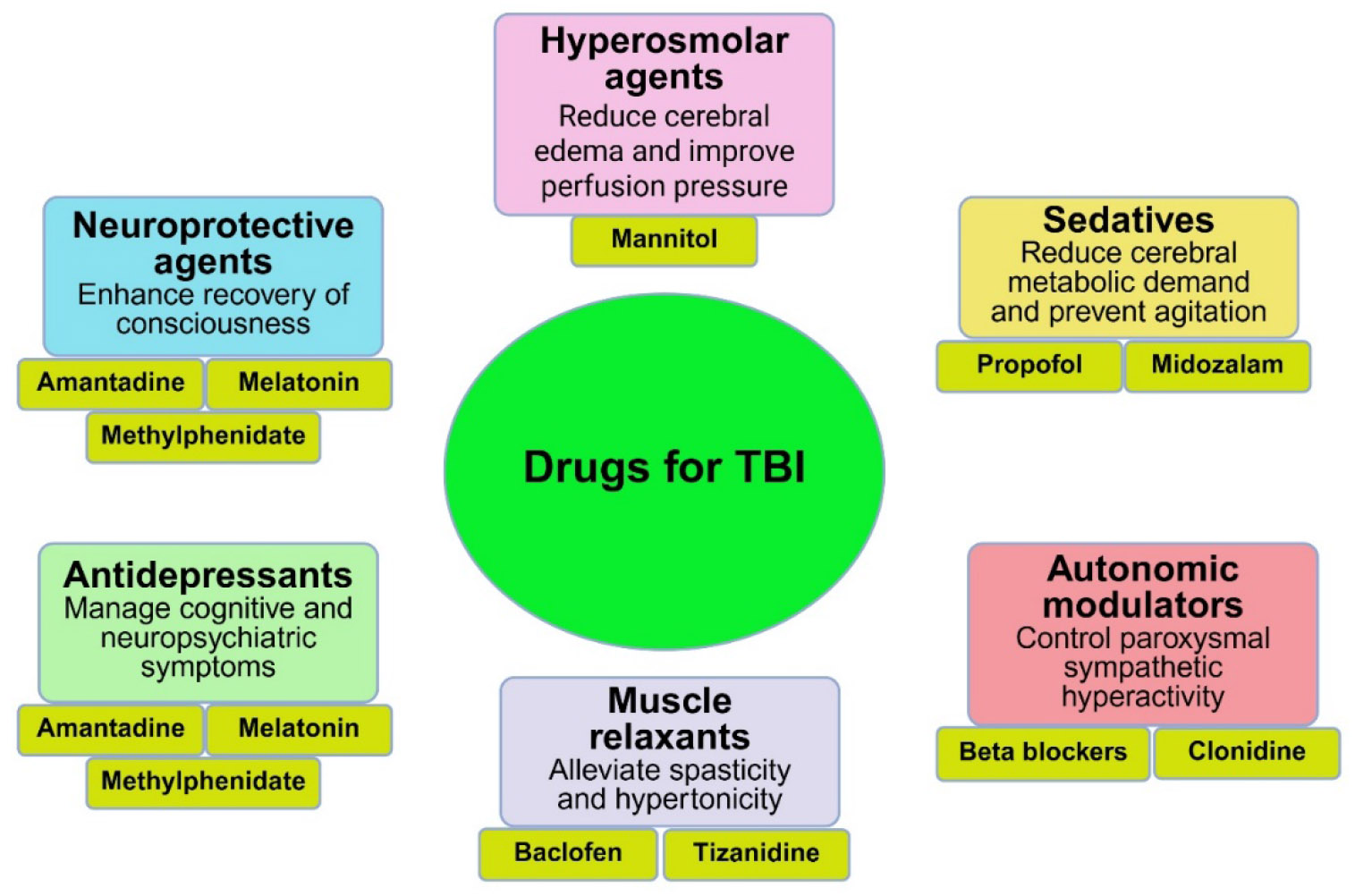
Categories of allopathic drugs used in the management of traumatic brain injury (TBI).

Traumatic brain injury (TBI) is a leading neurological disorder resulting from external mechanical forces such as impact, acceleration, or blast injuries [52]. It presents a spectrum of clinical manifestations, ranging from mild concussion to severe brain damage, and is associated with substantial morbidity and mortality globally. Allopathic treatment strategies for TBI are multifaceted and aim to prevent secondary injury, promote neuroprotection, and manage long-term cognitive, motor, and psychiatric sequelae (Figure 5). In the acute phase, the priority is stabilization of vital functions using Advanced Trauma Life Support (ATLS) protocols. Airway protection, ventilation, and hemodynamic stabilization are critical. Intracranial pressure (ICP) management is central to initial care, with hyperosmolar agents such as mannitol and hypertonic saline commonly used to prevent cerebral herniation and further neuronal injury. These agents reduce cerebral edema and improve perfusion pressure, a determinant of outcomes in severe TBI cases [53].

Sedation and analgesia using agents such as propofol and midazolam reduce cerebral metabolic demand and prevent agitation, which can worsen intracranial hypertension. In cases of refractory ICP elevation, therapeutic options include induced barbiturate coma and decompressive craniectomy. Antiepileptic drugs (AEDs), especially levetiracetam and phenytoin, are often administered prophylactically in the first week post-injury to reduce early post-traumatic seizures, although their role in preventing long-term epilepsy remains inconclusive [54]. Pharmacological neuroprotection has received increasing attention, particularly in the subacute and chronic phases of TBI (Figure 5). Agents such as amantadine, an NMDA receptor antagonist with dopaminergic activity, have shown benefits in enhancing recovery of consciousness and functional outcomes in moderate to severe TBI [55]. Methylphenidate, commonly used in attention-deficit disorders, is employed to improve attention span and cognitive processing speed in TBI patients experiencing cognitive fatigue [56]. Similarly, melatonin has been studied for its antioxidant and anti-inflammatory properties in reducing neuronal damage and enhancing post-injury neurocognitive function [57].

The inflammatory cascade following TBI, characterized by microglial activation, cytokine release, and disruption of the blood-brain barrier, presents another therapeutic target. While corticosteroids such as methylprednisolone were historically used to blunt neuroinflammation, large-scale trials like CRASH have revealed increased mortality associated with their use, leading to a shift away from corticosteroid-based therapies. Research is now focused on specific cytokine inhibitors and biologics that modulate immune responses without systemic immunosuppression [58,59]. Cognitive and neuropsychiatric symptoms following TBI—including depression, emotional dysregulation, agitation, and anxiety—are often managed with selective serotonin reuptake inhibitors (SSRIs) such as sertraline and escitalopram [60,61].

Recent research has explored novel pharmacologic agents for TBI management. Statins have shown anti-inflammatory and endothelial stabilizing effects in animal models, with early-phase human trials suggesting potential benefits in reducing cerebral edema [62]. Progesterone and erythropoietin, though promising in preclinical studies for neuroprotection and myelin repair, have failed to demonstrate efficacy in large randomized controlled trials [63]. Cannabinoids, particularly cannabidiol (CBD), are being investigated for their potential to reduce anxiety, neuroinflammation, and seizure risk, although robust clinical data in TBI populations remain scarce [64].

In post-acute and chronic stages, pharmacotherapy is complemented by comprehensive neurorehabilitation. Botulinum toxin A injections are used to manage focal spasticity, while oral muscle relaxants like baclofen and tizanidine alleviate central hypertonicity. Beta-blockers and clonidine may be used to control paroxysmal sympathetic hyperactivity, a syndrome of autonomic instability seen in some severe TBI cases. In summary, the allopathic approach to TBI incorporates a range of evidence-based interventions aimed at stabilizing acute injury, reducing secondary damage, and managing long-term complications. While no single pharmacologic agent has demonstrated universal efficacy in reversing TBI pathology, a symptom-targeted, multimodal strategy integrating pharmacological and rehabilitative interventions remains the current standard. Ongoing research into inflammatory modulators, neurostimulants, and regenerative therapies may further enhance recovery and quality of life in individuals living with TBI.

## 5. Phytochemicals That May Help in Neurological Diseases

**Figure 6:**
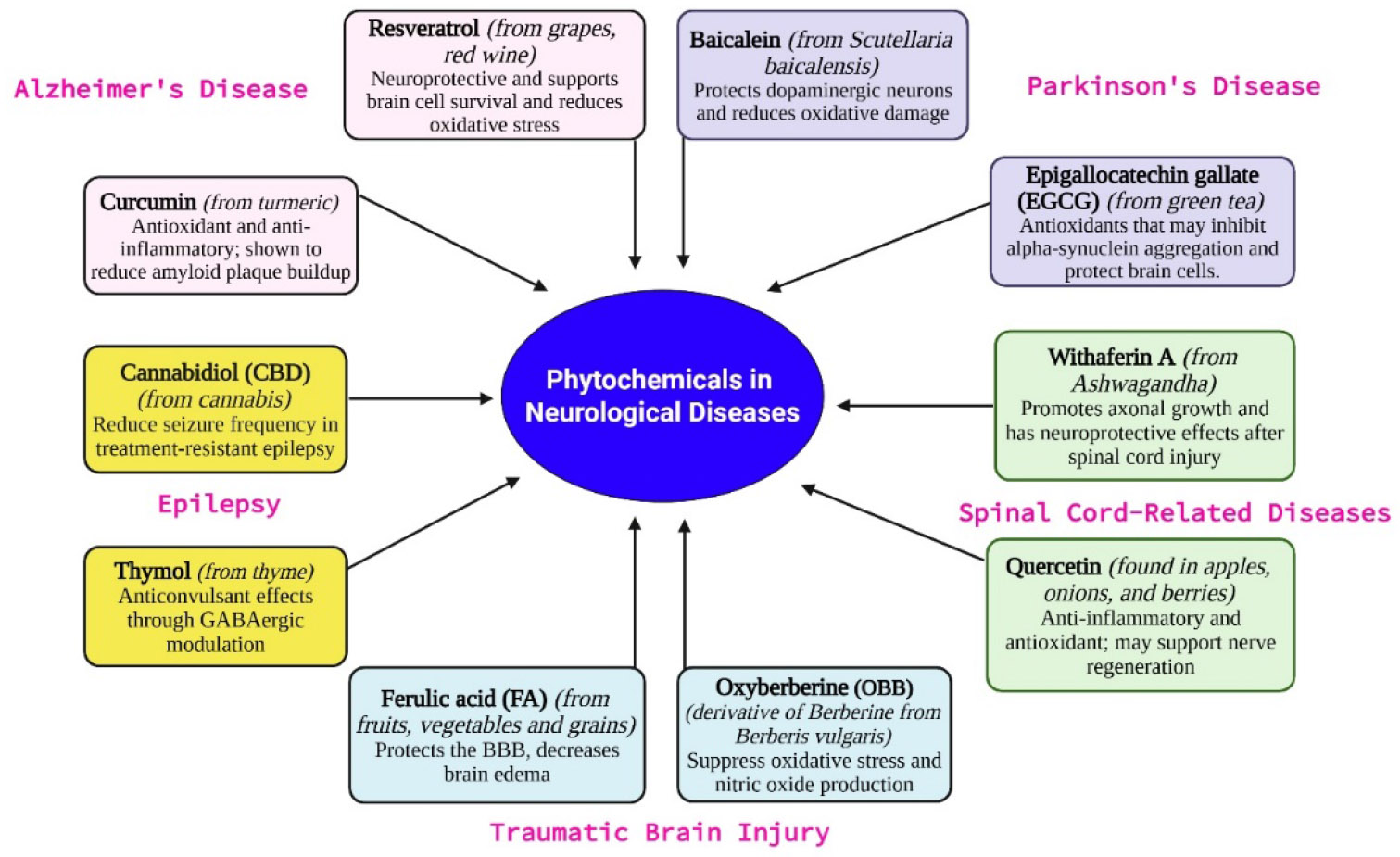
Classification of phytochemicals commonly used in the management of neurological diseases.

Phytochemicals—bioactive compounds derived from plants—have gained considerable attention for their potential therapeutic effects in neurological disorders. Many of these natural compounds exhibit antioxidant, anti-inflammatory, neuroprotective, and cognitive-enhancing properties. As conventional treatments often focus on symptom management, phytochemicals offer promising complementary or alternative strategies for targeting the underlying mechanisms of neurological diseases (Figure 6).

### 5.1 Alzheimer’s disease

Phytochemicals—naturally occurring plant-based compounds—have emerged as promising multi-target agents in the prevention and management of AD. Unlike synthetic drugs that largely provide symptomatic relief, phytochemicals exert disease-modifying effects by acting on key pathological hallmarks such as amyloid-β (Aβ) aggregation, tau hyperphosphorylation, neuroinflammation, oxidative stress, mitochondrial dysfunction, and synaptic loss. These compounds are chemically diverse and can be broadly categorized into polyphenols, alkaloids, terpenoids, and volatile phytochemicals.

#### 5.1.1 Polyphenols

Resveratrol, a stilbene polyphenol found in grapes and red wine, exhibits antioxidant, anti-inflammatory, and anti-amyloid effects by modulating signaling pathways involved in Aβ aggregation and tau phosphorylation. A phase II clinical trial confirmed its efficacy in reducing cerebrospinal fluid biomarkers of neuronal injury and inflammation in AD patients [65,66]. Its analog polydatin, with superior bioavailability, showed cognitive and biochemical improvements in aluminum chloride-induced AD rat models [67].

To improve delivery, chitosan-coated bovine serum albumin nanoparticles (CS-RES-BSANPs) were developed for intranasal administration, demonstrating enhanced brain penetration and behavioral outcomes [68]. Similarly, dihydro-resveratrol (DHR), a microbial metabolite, improved cognition in AD mouse models by enhancing mitophagy via the ULK1-Bnip3 pathway [69].

Another key polyphenol, Curcumin, the main bioactive compound in *Curcuma longa* (turmeric), has demonstrated potent antioxidant, anti-inflammatory, anti-amyloid (Aβ), and anti-tau properties in Alzheimer’s disease (AD) models [70]. To address its low solubility and poor bioavailability, several nanocarrier-based delivery systems have been developed. These include MWCNT-COOH-PEG, which enhances brain targeting and provides sustained release [71]. curcumin-silver nanoparticles with notable acetylcholinesterase inhibition and antioxidant activity [72]. BP-PEG-Tar@Cur, which reduces Aβ aggregation and oxidative stress in AD mice [73]. C3/TPP-EXO-CUR, an engineered exosome system targeting damaged mitochondria to lower tau phosphorylation and neuronal apoptosis [74], and EXO-Cur+MB, a co-delivery exosomal system with methylene blue that effectively inhibits tau hyperphosphorylation via the AKT/GSK-3β pathway [75].

Further strategies included curcumin-enriched mesenchymal stem cell exosomes (CUR-MSC-EXO), which modulated microglial polarization and improved memory [76], and curcumin-selenium nanoemulsions, which reduced oxidative stress and tau/Aβ burden [77]. Diagnostic approaches using [18F]-DiFboron-8, a PET imaging probe derived from half-curcumin, enabled non-invasive Aβ plaque detection [78], while ophthalmoscopy using curcumin contrast identified retinal Aβ deposits, aiding early AD diagnosis [79]. A chemically modified curcumin derivative, Derivative 27, activated the Nrf2 pathway and significantly reduced hippocampal Aβ burden and inflammation, demonstrating enhanced efficacy at lower doses [80].

Quercetin, a flavonoid abundant in apples, onions, and leafy greens, exerts neuroprotective effects through oxidative stress inhibition, tau dephosphorylation, and anti-inflammatory activity. Encapsulated as nanostructured lipid carriers (QC-NLCs), quercetin improved brain uptake and decreased Aβ deposition in rat models [81]. It also inhibited early stress-induced AD pathology via NLRP3 inflammasome modulation [82]. and acted synergistically with donepezil to reduce tau and BACE1 expression while restoring miRNA-124 levels [83]. Additionally, quercetin modulated P2X7 receptors, implicated in ATP-mediated neuroinflammation [84]. To enhance gut delivery and systemic efficacy, quercetin-decorated selenium nanoparticles (QUE@SeNPs) within chitosan/PVP nanofibers were developed. This system improved gut–liver–brain axis regulation and cognitive outcomes through microbiota modulation and gut barrier restoration [85].

Another promising polyphenol, Dip-1 from *Dipteris wallichii*, was identified as a potent BACE1 inhibitor, exhibiting high BBB permeability and a low IC_50_ value, supporting its role in Aβ pathology suppression [86].

#### 5.1.2 Alkaloids

Berberine (BBR), an isoquinoline alkaloid extracted from *Berberis vulgaris*, offers neuroprotection through dual mechanisms: central anti-inflammatory and anti-amyloid effects, and peripheral modulation of the gut–brain axis. In AD models, it reduced Aβ plaques, enhanced spatial memory, and improved gut microbiota diversity and barrier function [87]. Importantly, berberine reversed D-ribose-induced cognitive impairment by demethylating the PINK1 promoter, restoring mitophagy via the PINK1–Parkin pathway [88]. A meta-analysis of 19 animal studies confirmed its efficacy in improving cognition and downregulating APP expression [89].

#### 5.1.3 Terpenoids

Ginsenoside Rb1, a triterpenoid saponin from *Panax ginseng*, has shown therapeutic efficacy in reducing Aβ burden, tau hyperphosphorylation, oxidative stress, and neuronal apoptosis. It modulates apoptotic signaling by decreasing Bax and cleaved caspase-3 while increasing Bcl-2 expression, leading to improved cognitive and memory functions in AD models [90,91]. Ginkgolide, a diterpenoid lactone from *Ginkgo biloba*, demonstrated anti-inflammatory and anti-amyloid properties. In APP/PS1 mice, it attenuated Aβ accumulation, reduced activation of the NLRP3 inflammasome, and lowered pro-inflammatory cytokines such as IL-1β and ROS, underscoring its potential in halting AD-associated neuroinflammation [92].

#### 5.1.4. Volatile Phytochemicals and Lignans

Several volatile compounds exhibit anti-amyloid and anti-tau actions. Cinnamaldehyde, phenylethyl alcohol, α-asarone (ASA), and β-caryophyllene (BCP), sourced from aromatic plants, prevented Aβ(25–35) fibrillation and reduced β-sheet content, as confirmed by spectroscopic and microscopic analyses [93]. ASA and BCP also disassembled pre-formed tau fibrils and protected neuroblastoma cells against tau-induced toxicity [94].

Among lignans, secoisolariciresinol diglucoside (SDG) from flaxseed demonstrated cognitive benefits in transgenic female AD mice. SDG suppressed Aβ deposition and neuroinflammation, enhanced GPER-CREB/BDNF signaling, and modulated gut microbiota, linking its action to gut-brain communication [95].

### 5.2 Parkinson’s Related Phytochemicals

These compounds target various pathological mechanisms of PD including oxidative stress, mitochondrial dysfunction, α-synuclein aggregation, and neuroinflammation. This section outlines key classes of phytochemicals that show promise in PD management, focusing on specific compounds and their mechanisms of action.

#### 5.2.1 Flavonoids

Flavonoids, a major class of phytochemicals, exhibit neuroprotective effects primarily through antioxidant and anti-inflammatory mechanisms. Baicalein, derived from *Scutellaria baicalensis* (Chinese skullcap), targets apoptotic and oxidative stress-related pathways. It interacts with key proteins such as MAPK1, EP300, and CREBBP [96], and in a 6-hydroxydopamine (6-OHDA) PD model, activates mitochondrial autophagy via the miR-30b-5p/SIRT1/AMPK/mTOR axis, reducing neuronal apoptosis [97]. Its metabolite baicalin also exerts neuroprotection by activating Nrf2 and suppressing NLRP3 inflammasome signaling in both in vitro (α-syn/MPP^+^) and in vivo (MPTP) PD models [98].

Epigallocatechin gallate (EGCG), a catechin from green tea, protects dopaminergic neurons through antioxidant and mitochondrial-stabilizing effects [99]. In 6-OHDA-induced SK-N-AS cells, it enhances viability, inhibits caspase-3, and reduces IL-1β [100]. Liposomal EGCG formulations (PC/PS-based, vitamin E-coated) improved uptake and suppressed LPS-induced neuroinflammation in PD rats [101], while in rotenone-induced models, EGCG preserved neurotransmitter levels and mitochondrial integrity [102].

Quercetin, found in apples, onions, and cocoa husk (*GuaCa extract*), alleviates motor and non-motor symptoms in PD. It reduces hippocampal IL-6, preserves dopaminergic neurons, enhances BDNF, and activates PI3K/Akt/GSK-3β signaling [103-105]. Nanoformulations, such as QAE NPs and quercetin-loaded niosomes, facilitate intranasal delivery, improving mitochondrial homeostasis and M2 microglial polarization [106,107]. Additionally, quercetin inhibits NLRP3 inflammasome activation *in vitro* (e.g., from *Cola acuminata*) [108].

Apigenin, from *Campsis grandiflora*, promotes chaperone-mediated autophagy, clears α-synuclein aggregates, and activates Nrf2. It modulates PI3K/Akt/NF-κB pathways and reduces caspase-3 activity [109,110]. In MPTP-induced mice, apigenin normalized cytokine profiles and ameliorated histological damage [111]. Docking studies confirm its binding affinity to PD-relevant targets including α-synuclein [112].

Rutin, a glycoside flavonoid found in *Berula erecta* and other sources, improves motor and gastrointestinal functions via modulation of enteric glial reactivity and nitric oxide signaling [113]. It also restores neurotransmitter balance and cognitive function in rotenone-induced models [114].

#### 5.2.2 Polyphenols and Stilbenes

Resveratrol, a stilbene from grapes and berries, enhances lifespan, motor function, and oxidative balance in transgenic Drosophila expressing α-synuclein [115]. In MPP^+^-treated SH-SY5Y cells, resveratrol-loaded neural stem cell-derived exosomes (RES-hNSCs-Exos) restored mitochondrial function and suppressed NLRP3 via AMPK-Nrf2 activation [116]. Leptin/transferrin-decorated nanoparticles co-loaded with resveratrol and ceftriaxone targeted dopaminergic neurons and modulated pathways like MAPK/ERK and α-synuclein [117]. Solid lipid nanoparticle microneedle patches improved resveratrol bioavailability, antioxidant capacity, and behavior without skin irritation [118]. In A53T PD mice, resveratrol restored mitochondrial VDAC1 function, reducing α-synuclein–VDAC1 interaction and calcium imbalance [119].

Oligomeric stilbenes (OSs) from *Alpha grape stems* (e.g., vitisin A, trans-vitisin B) showed antioxidant neuroprotection *in vitro* [120], while α-viniferin, a resveratrol trimer, exhibited potent MAO inhibition, dopamine enhancement, and motor improvement without toxicity in Drosophila and murine models [121].

#### 5.2.3 Alkaloids

Alkaloids, a diverse class of nitrogen-containing phytochemicals, have shown promising neuroprotective potential in Parkinson’s disease (PD) by modulating neurotransmission, inhibiting neuroinflammation, and supporting neuroplasticity. Harmine and harmaline, alkaloids from *Peganum harmala*, act as MAO-A inhibitors, increasing serotonin, dopamine, and noradrenaline levels. They also activate Nrf2, suppress NF-κB, and enhance BDNF/TrkB, improving PD-related behavioral and oxidative parameters in stress-induced rats [122].

Humulus japonicus water extract (HJW) countered scopolamine-induced cognitive decline by inhibiting acetylcholinesterase and activating CaMKIIα-CREB and AKT-GSK3β pathways, supporting neuroplasticity [123]. Although synthetic, PZKKN-94, a 5-HT1B agonist/5-HT6 antagonist, mimics phytochemical serotonergic activity seen in agents like resveratrol and curcumin, providing neuroprotection, antidepressant-like effects, and cognitive enhancement in PD models [124]. A blend of safflower seeds (*Carthamus tinctorius*) and dandelion (*Taraxacum coreanum*) extracts (CTS-TC) improved cognition and reduced neuroinflammation in scopolamine-treated models. HPLC identified active constituents including N-feruloylserotonin, chicoric acid, and chlorogenic acid with antioxidant and neuromodulatory properties [125].

#### 5.2.4 Phenolic Acids

Phenolic acids, especially hydroxycinnamic acids, target α-synuclein aggregation and neuroinflammation. Sinapic acid and chlorogenic acid inhibit α-synuclein fibrillation and suppress its β-sheet conversion, even post-aggregation, demonstrating utility at various PD stages [126]. Ferulic acid (FA), found in grains and fruits, improves dopaminergic survival, mitochondrial gene expression, TH levels, and reduces α-synuclein, oxidative stress, and NF-κB activity in MPTP and rotenone-induced models [127,128]. Caffeic acid, abundant in *Brassica juncea* and *Eucommia ulmoides*, preserves dopaminergic neurons and regulates autophagy (e.g., LC3b, ATG7, α-syn) through 4E-BP1 activation [129]. Embedded in hydrogels like OACDP, it offers controlled delivery and mitigates neuroinflammation and oxidative injury [130]. Caffeic acid-derived carbon quantum dots (CACQDs) protect against paraquat-induced toxicity via free radical scavenging and anti-aggregation properties [131]. Synthetic phenolic derivatives based on proline and GABA scaffolds show multitarget effects, including lipid peroxidation inhibition, anti-inflammatory activity, and mild acetylcholinesterase inhibition—relevant to PD pathology [131].

### 5.3 Epilepsy-Related Phytochemicals

Phytochemicals offer promising alternatives for managing epilepsy, particularly drug-resistant forms, through diverse mechanisms including modulation of neurotransmission, anti-inflammatory activity, antioxidant defense, and gut-brain axis regulation. These compounds are broadly classified into cannabinoids, monoterpenes, alkaloids, flavonoids, polyphenols, and saponins.

#### 5.3.1 Cannabinoids

Cannabidiol (CBD), a non-psychoactive compound from *Cannabis sativa*, is FDA- and EMA-approved for seizures in tuberous sclerosis and under investigation for broader neuropsychiatric symptoms [133]. Multiple preclinical studies reinforce CBD’s anticonvulsant efficacy. Preclinical models (e.g., zebrafish, SE) show its anticonvulsant effects and glutamate modulation [134,135]. Clinically, CBD significantly reduces seizure frequency in drug-resistant epilepsy (DRE), with up to 50% responder rates, though higher doses may cause side effects [136-138]. Mechanistically, CBD suppresses reactive astrocytes and neuroinflammation, while pharmacokinetic studies highlight interindividual variability and the need for liver function monitoring [139,140]. Therapeutic drug monitoring (TDM) studies reveal significant pharmacokinetic variability in CBD and its metabolite 7-hydroxy-CBD, emphasizing the need for individualized dosing and liver function monitoring due to moderate ALT elevations [140]. A review of 47 clinical trials confirmed CBD’s anticonvulsant effects, particularly in Dravet and Lennox-Gastaut syndromes, while stressing the need for standardized dosing and trial protocols [141]. Additionally, non-cannabis analogs such as *Magnolia spp.*-derived magnolol and honokiol, and amorfrutin 2 from *Amorpha fruticosa*, inhibit T-type calcium channels, offering cannabinoid-independent anticonvulsant activity [142].

#### 5.3.2 Monoterpenes

Monoterpenes, volatile compounds primarily found in essential oils, exhibit strong anticonvulsant and neuroprotective effects. (+)-Borneol, from *Dryobalanops aromatica*, enhanced the potency and brain levels of retigabine, significantly reducing required doses in resistant seizure models [143]. Geraniol, present in *Cymbopogon* and *Pelargonium*, provided protection via GABAergic enhancement, oxidative stress reduction, and cytokine modulation [144]. Alpha-pinene, a major constituent of *Pinus* species, attenuated seizures in kainic acid models by downregulating NF-κB and ERK1/2 signaling and reducing glial activation [145].

#### 5.3.3 Alkaloids

Alkaloids are nitrogen-containing compounds known for potent neuroactivity. Stachydrine, from *Leonurus japonicus*, showed dual inhibition of Notch1 and NMDA receptor pathways in PTZ-induced mice, reducing excitotoxicity, inflammation, and cognitive decline [146]. Berberine (BBR), from *Berberis vulgaris*, alleviated hippocampal damage and modulated the gut-brain axis by increasing beneficial microbiota and altering SCFA and brain lipid profiles, highlighting microbiome-mediated anticonvulsant pathways [147].

#### 5.3.4 Flavonoids

Flavonoids, abundant in fruits and herbs, act via antioxidant, anti-inflammatory, and GABAergic pathways. Quercetin, found in onions and apples, reduced seizure duration and preserved antioxidant enzymes when delivered via chitosan nanoparticles [148]. Extracts from *Cyanthillium cinereum*, rich in quercetin, kaempferol, and gallic acid, delayed seizure onset and improved oxidative status [149].

Luteolin, from *Salvia miltiorrhiza*, inhibited apoptosis and inflammation through MAPK/NF-κB and GADD45B targeting in kainic acid models [150]. Glabranin and 3'-hydroxy-4'-O-methylglabridin from *Glycyrrhiza glabra* were identified via AI-guided modeling as AKT1 inhibitors [151]. Kaempferol, from tea and broccoli, showed high brain delivery via intranasal phospholipid magnesomes and sustained anti-seizure effects [152]. Apigenin, present in parsley and chamomile, raised seizure thresholds by increasing GABA-A receptor expression [153]. Naringin, a citrus flavonoid, reduced seizures and cognitive deficits by suppressing HMGB1-TLR4 signaling and enhancing neuroprotective factors like Klotho and ADAM-10 [154].

#### 5.3.5 Polyphenols

Polyphenols offer antioxidant and anti-inflammatory benefits in seizure control. Caffeic acid, abundant in coffee, reduced seizures and neuroinflammation by inhibiting the PERK–NF-κB pathway via aconitate decarboxylase 1 binding [155]. In *Origanum majorana*, caffeic acid and quercetin delayed seizures through NMDA receptor interaction [156]. Curcumin and its analog mitocurcumin (MitoCur) reduced oxidative stress in zebrafish seizure models, with MitoCur displaying higher efficacy at low doses but pro-oxidant effects at higher levels [157]. Wogonin, from *Scutellaria baicalensis*, restored synaptic density and suppressed microglial overactivation via AKT/FoxO1 signaling [158]. Lippia multiflora, rich in polyphenols and flavonoids, improved seizure outcomes and normalized TNF-α, IL-1β, and IL-6 levels in chronic epilepsy models [159].

#### 5.3.6 Saponins

Saponins, particularly steroidal and triterpenoid forms derived from various medicinal plants, have shown significant antiepileptic potential by modulating neuronal excitability, neurotransmission, mitochondrial function, and inflammation.

From *Solanum torvum*, steroidal saponins like torvosides A, X, Y showed strong activity in zebrafish via glycosylation at C-6 [160]. Saponins from *Anemarrhena asphodeloides* (AAS) mitigated hippocampal neuron loss and downregulated epilepsy-related proteins HSP90AB1 and YWHAB [161]. Ginsenoside Re (GRe) from *Panax ginseng* reduced mitochondrial oxidative stress and seizures, partly via IL-6/STAT3 signaling [162]. Diosgenin, from *Dioscorea spp.*, improved gut microbiota, reduced glial activation, and modulated TLR4-MyD88 signaling, linking its effect to gut–brain axis regulation [163]. Saikosaponin-a (SSa), from *Bupleurum spp.*, encapsulated in MePEG-PCL nanoparticles, enhanced brain delivery and significantly reduced seizures, neuronal apoptosis, and hippocampal damage while increasing GABA-A receptor levels [164].

### 5.4 Spinal Cord-Related Phytochemicals

SCI is characterized by an initial mechanical insult followed by a cascade of secondary injuries such as inflammation, oxidative stress, apoptosis, and autophagy, which worsen neuronal damage. Certain phytochemicals have demonstrated neuroprotective effects by modulating these secondary injury pathways.

#### 5.4.1 Flavonoids

Flavonoids are plant-derived polyphenolic compounds known for their potent antioxidant, anti-inflammatory, and neuroprotective effects. Several studies have highlighted their therapeutic potential in managing spinal cord injury (SCI) and related neuropathies.

Corn silk extract (CSE) from *Zea mays* stigmas contains flavonoids such as maysin, apigenin, and luteolin. It demonstrated neuroprotective effects in paclitaxel-induced peripheral neuropathy (PIPN) by reducing oxidative stress, inflammation, and overactivation of the renin–angiotensin system. CSE preserved spinal cord and sciatic nerve structure, reduced demyelination, and downregulated NF-κB and ACE activity, supporting its use in multi-targeted neuropathic treatment [165].

Catechin, found in green tea (*Camellia sinensis*), cocoa, and fruits, showed efficacy in a chronic constriction injury model by reducing mechanical hyperalgesia and suppressing neuroinflammation. It inhibited iNOS, COX-2, and pro-inflammatory cytokines, while promoting an anti-inflammatory M2 microglial phenotype via suppression of the TLR4/MyD88/NF-κB and MAPK pathways [166].

Calycosin, an isoflavone from *Astragalus membranaceus*, mitigated glial scar formation in SCI by inhibiting A1 astrocyte activation. It reduced complement C3 expression and STAT3 phosphorylation, suggesting its regulatory effect on astrocyte-mediated inflammation [167].

Quercetin, widely present in *Allium cepa*, *Malus domestica*, and *Camellia sinensis*, inhibited ferroptosis in oligodendrocyte progenitor cells by blocking NCOA4-mediated ferritinophagy. It preserved mitochondrial integrity, reduced lipid peroxidation, and maintained iron homeostasis, indicating its protective role in SCI [168]. Quercetin also showed antinociceptive effects in a spinal cord hemi-contusion model when derived from *Trifolium resupinatum* essential oil, through modulation of NO-cGMP-K^+^ pathways and TRPV1/CB1 receptor interactions, along with suppression of inflammatory cytokines [169].

Baicalin, extracted from *Scutellaria baicalensis*, exerted anti-inflammatory effects in SCI by binding the TLR4/MD2 complex on microglia and inhibiting PI3K/AKT/NF-κB signaling. It reduced glial activation and indirectly suppressed astrocyte-mediated inflammation by decreasing TNF-α, IL-1α, and C1q levels [170].

Rutin, present in *Fagopyrum esculentum*, apples, and citrus fruits, improved locomotor recovery and reduced neuroinflammation in a distraction spinal cord injury model. It inhibited the P38 MAPK/NF-κB/STAT3 axis and attenuated neuronal apoptosis and demyelination. Molecular docking confirmed its interaction with MAPK13 [171].

Rosmarinic acid, a polyphenolic compound from *Rosmarinus officinalis*, *Ocimum basilicum*, and *Origanum vulgare*, promoted sciatic nerve regeneration after taxol-induced injury. It inhibited NF-κB signaling, enhanced CGRP expression and axon number, although behavioral outcomes remained unchanged [172].

#### 5.4.2 Alkaloids

Alkaloids, nitrogen-containing secondary metabolites commonly derived from medicinal plants, have demonstrated significant neuroprotective potential in spinal cord injury (SCI) through their antioxidant, anti-inflammatory, and anti-ferroptotic properties.

**Aloperine**, a quinolizidine alkaloid from *Sophora spp.*, improved locomotor function and preserved spinal tissue in a rat contusion SCI model. It reduced markers of apoptosis (Bax, cleaved caspase-3), oxidative stress (4HNE, MDA), and inflammation (NF-κB, TNF-α), while upregulating antioxidant enzymes (SOD1, GPx1) through activation of PI3K/AKT and inhibition of NF-κB pathways [173].

Berbamine, extracted from *Stephania epigaea*, alleviated neuropathic pain in a chronic constriction injury model by inhibiting the TMEM34/SGK1/FOXO3 axis, thus reducing astrocyte and neuron activation and behavioral signs of allodynia and hyperalgesia [174]. **Tetrahydroberberine (THB)**, derived from *Corydalis spp.* (family Papaveraceae), significantly improved motor recovery and tissue repair in SCI mice by activating the Nrf2 pathway, reducing lipid peroxidation, and inhibiting ferroptosis. The protective effects were negated upon Nrf2 inhibition, confirming pathway involvement [175].

Tomatidine, a steroidal alkaloid from *Solanum lycopersicum* (tomato), promoted neuronal recovery, reduced apoptosis and inflammation in vivo and in vitro, and exerted its effects by downregulating CXCL10 and inhibiting the NF-κB pathway [176].

**Tetrandrine,** a bis-benzylisoquinoline alkaloid from *Stephania tetrandra*, showed neuroinflammatory modulation in SCI therapy. Delivered via MPEG-PDLLA nanoparticles embedded in GelMA microgels, it suppressed neurotoxic glial crosstalk and enhanced spinal repair post-injection [177].

**Tetramethylpyrazine (TMP),** isolated from *Ligusticum chuanxiong*, improved motor function and reduced neuronal death in SCI by inhibiting ferroptosis. TMP activated the NRF2/ARE pathway to regulate antioxidant gene expression and maintain iron homeostasis [178].

**Berberine (BBR)**, an isoquinoline alkaloid from *Berberis vulgaris*, mitigated SCI-induced ferroptosis by reducing lipid peroxidation, iron accumulation, and mitochondrial dysfunction. It activated the AMPK-NRF2-HO-1 pathway, and inhibition of AMPK reversed its protective effects, confirming mechanistic specificity [179].

#### 5.4.3 Polyphenols

Polyphenols, widely distributed in plants, possess strong antioxidant, anti-inflammatory, and neuroprotective properties, making them valuable therapeutic agents in spinal cord injury (SCI).

**Epigallocatechin gallate (EGCG),** a major catechin derived from *Camellia sinensis* (green tea), has demonstrated neuroregenerative effects in SCI models. EGCG reduced oxidative stress by activating the Keap1/Nrf2/HO-1 pathway and upregulating antioxidant enzymes such as SOD1 and SOD2. Incorporated into stem cell sheets or fibrin scaffolds, EGCG enhanced cell viability, promoted angiogenesis and axonal regeneration, and improved functional outcomes in rats with SCI [180,181].

**Resveratrol,** a stilbene polyphenol found in *Vitis vinifera* (grape), *Arachis hypogaea* (peanut), and various berries, has been extensively studied for SCI therapy. In exosome-based delivery systems, resveratrol targeted inflamed spinal tissues, inhibited reactive oxygen species (ROS), reduced glial scarring, and modulated A1 astrocyte activity [182]. Resveratrol also reduced pyroptosis by upregulating miR-124-3p and downregulating DAPK1, thereby inhibiting the NLRP3/caspase-1/GSDMD pathway [183]. Additionally, it improved the anti-inflammatory effects and survival of bone marrow mesenchymal stem cells (BM-MSCs) by activating SIRT1 and suppressing NF-κB, resulting in enhanced neuroprotection and motor recovery [184]. Combined with calcium, resveratrol further showed promise in preventing SCI-induced osteoporosis through SIRT1/FOXO3a signaling, improving bone microarchitecture and resistance to fracture [185]. Moreover, polyphenolic extracts from grape stalks and *Coffea arabica* (coffee) reduced neuropathic pain and modulated neuroimmune pathways (CCL2/CCR2, CX3CL1/CX3CR1) in supraspinal regions, reducing glial activation and behavioral hypersensitivity in mice [186].

**Curcumin,** a polyphenol from *Curcuma longa* (turmeric), has shown diverse neuroprotective roles in SCI. When embedded in polydopamine-based scaffolds, curcumin attenuated inflammation and oxidative stress by inhibiting NF-κB and promoting JAK/STAT signaling, thus enhancing axonal regeneration [187]. It also reduced neuropathic pain, improved motor function, and increased antioxidant markers (SOD, catalase, GABA-A) while lowering lipid peroxidation and tissue damage in SCI rats [188]. A network pharmacology study identified matrix metalloproteinase-9 (MMP9) as a critical curcumin target, with curcumin downregulating its expression and conferring protection in cellular SCI models [189].

### 5.5 Traumatic Brain Injury

Traumatic brain injury (TBI) remains a critical cause of disability and mortality worldwide, leading to cognitive, emotional, and motor dysfunction [190]. While no definitive pharmacological therapy exists to reverse secondary brain damage, a growing body of evidence supports the role of phytochemicals as neuroprotective agents. These natural compounds, derived from plants, exhibit anti-inflammatory, antioxidant, anti-apoptotic, and neurogenic properties. Here, we explore the classification and mechanisms of action of key phytochemicals studied in the context of TBI.

#### 5.5.1 Polyphenols

Polyphenolic compounds, particularly curcumin and resveratrol, exhibit strong neuroprotective effects in TBI by modulating oxidative stress, inflammation, apoptosis, and autophagy. Advanced formulations and derivatives enhance their bioavailability and therapeutic outcomes.

Curcumin, a major turmeric polyphenol, offers multifaceted neuroprotection in TBI by targeting oxidative stress and neuroinflammation. Nanoparticle formulations like CAQK-modified C-PPS/C enhanced brain targeting, reduced ROS and NF-κB activity, preserved BBB integrity, and improved neurological recovery [191]. Preventive use of turmeric extract in repetitive TBI reduced TNF-α, GFAP, p-Tau, and TDP-43 levels [192]. Derivatives such as **bisdemethoxycurcumin (BDMC)** and **tetrahydrocurcumin (THC)** further modulated autophagy and microglial polarization through the HSP90AA1/TFEB/Nrf2 and GSK3β/PTEN/PI3K/Akt pathways, respectively, reducing inflammation and apoptosis.

Resveratrol (RSV) also shows strong efficacy in TBI. Functionalized silver nanowire MXene biopatches combining RSV enabled real-time GFAP monitoring and reduced inflammation [195]. RSV downregulated ER stress proteins (CHOP, GRP78), pro-inflammatory cytokines (TNF-α, IL-1β), and oxidative stress markers (MDA), while upregulating GSH and maintaining hippocampal architecture [196,197].

#### 5.5.2 Terpenoids

**Withaferin A (WFA)** from *Withania somnifera* improved neurobehavior, reduced BBB disruption, edema, and endothelial apoptosis, while decreasing IL-1β, IL-6, TNF-α and microglial activation [198]. **Ginkgolide A (GA)** from *Ginkgo biloba* enhanced neurological function, decreased oxidative stress (MDA, 8-OHdG), and inhibited apoptosis, supported by increased SOD activity [199]. **Ginsenoside Rg3,** from ginseng, reduced brain edema, microglial inflammation, and hippocampal damage by activating SIRT1 and inhibiting NF-κB; its effect was blocked by SIRT1 inhibitor NAM, confirming pathway involvement [200].

#### 5.5.3 Alkaloids

Oxyberberine (OBB), a berberine derivative, formulated as nanocrystals (OBB-NC), attenuated neuroinflammation via HMGB1/TLR4/NF-κB pathway inhibition. It improved cognitive, anxiety, and depression-like behaviors post-TBI and suppressed oxidative stress and nitric oxide production [201].

#### 5.5.4 Flavonoids

**Quercetin**, a common dietary flavonoid, demonstrated broad neuroprotection by targeting oxidative stress, inflammation, ferroptosis, and apoptosis. Network pharmacology identified hub genes (e.g., HIF1A, IL6, TP53), and pathways (HIF-1, PI3K-Akt, IL-17) as key mediators [202]. *In vivo*, quercetin suppressed microglial activation, reduced edema and neuronal death, and acted via the PGC-1α/Nrf2 pathway while inhibiting HDAC3 nuclear translocation [203].

#### 5.5.5 Phenolic Acids

Ferulic acid (FA), a key component of traditional formulas like Guanxin II and DCH, modulated the gut-brain axis, reduced GI dysfunction, and suppressed neuroinflammation through Ghrelin/GHSR signaling and inhibition of the TLR4/NF-κB/NLRP3 pathway [204]. It also protected the BBB, decreased brain edema, and influenced HPA axis hormones including dopamine, serotonin, and BDNF, further validating its role in holistic neuroprotection [205].

## 6. Interactions between Phytochemicals and Allopathic Drugs

Natural compounds, including herbal medicines and dietary phyto-compounds, have increasingly been utilized for therapeutic synergy with conventional drugs used to treat various brain disorders. Such combinational approaches capitalize on antioxidant, anti-inflammatory, neuroprotective, and immune-boosting properties inherent to these natural substances. However, these combinations can also lead to unintended pharmacokinetic or pharmacodynamic interactions, significantly influencing therapeutic outcomes.

Flavonoids such as quercetin and silymarin are prominent for modulating transporter proteins like P-glycoprotein (P-gp), affecting the brain availability of several CNS drugs. High doses of quercetin inhibit P-gp, enhancing brain levels of medications like ritonavir and quinidine, while low doses may activate efflux mechanisms, decreasing uptake of agents such as vincristine [206-208]. Procyanidins from pine bark similarly inhibit P-gp at the blood-brain barrier (BBB), facilitating greater accumulation of chemotherapeutic drugs within brain tissues, which is particularly beneficial in managing brain tumors [209].

Curcumin, another polyphenol, notably boosts the delivery and efficacy of brain-targeted therapeutics when administered via nanoparticle systems. For example, curcumin-loaded nanoparticles significantly improve brain localization of doxorubicin, thereby enhancing its effectiveness against brain cancers through P-gp inhibition [210]. Additionally, curcumin demonstrates pharmacodynamic synergy with antiepileptic drugs, improving cognitive and neuroprotective outcomes without altering systemic drug levels [211].

Compounds like borneol, known for their membrane-permeabilizing properties, have been used to enhance CNS drug delivery. When incorporated into nanoparticles or niosomes, borneol significantly increases the brain uptake of drugs like dopamine, ginkgolide B, and puerarin, suggesting an improved therapeutic potential in models of Parkinson’s disease [212,213].

Terpenoids in Ginkgo biloba (GB) extracts also influence drug metabolism. GB has shown dual effects—enhancing midazolam exposure while reducing carbamazepine plasma levels—likely due to the interplay between its terpene lactones and flavonol glycosides on CYP3A4 activity [214,215].

Compounds such as hyperforin from St. John’s Wort (SJW) induce both CYP3A4 and P-gp expression, leading to reduced plasma concentrations of drugs like amitriptyline and docetaxel, and thus diminishing their therapeutic effects [216,217]. Variations in SJW extract composition further impact interaction severity, underlining the importance of standardized formulations.

In contrast, grapefruit juice, rich in furanocoumarins, inhibits CYP3A4, notably increasing plasma levels of carbamazepine, a common antiepileptic. This interaction can enhance therapeutic outcomes but also raises toxicity risks if not monitored carefully [218]. Ginsenosides—particularly protopanaxadiol derivatives from Ginseng—demonstrate both pharmacodynamic and pharmacokinetic interactions. Co-administration with carbamazepine in liver microsome studies showed enhanced CYP3A4-mediated metabolism, potentially reducing therapeutic exposure [219]. These findings suggest caution in the simultaneous use of Ginseng products with drugs primarily metabolized by CYP3A4. Imperatorin, found in Angelica sinensis and Angelica dahurica, enhances diazepam levels by inhibiting its hepatic metabolism, reducing clearance, and increasing systemic exposure [220]. Similar effects are seen with Khat (Catha edulis), a CNS stimulant, which inhibits both CYP3A4 and CYP2D6—enzymes critical for the metabolism of antipsychotics like aripiprazole and antidepressants such as clomipramine [221].

Melatonin exerts notable pharmacodynamic synergy with antiepileptic drugs such as carbamazepine and phenytoin. These effects are independent of melatonin's influence on drug plasma levels, pointing instead to its intrinsic neuroprotective and modulatory properties on neuronal excitability [222,223]. Caffeine, although widely consumed, may interfere with epilepsy management. It has been shown to diminish the anticonvulsant efficacy of several antiepileptics including carbamazepine, valproate, and topiramate, via pharmacodynamic mechanisms rather than alterations in systemic concentrations [224]. Quecertin stands out for its ability to boost the cytotoxic effects of doxorubicin and temozolomide in brain tumor models through inhibition of heat shock protein expression. This pharmacodynamic enhancement suggests promising avenues for adjunctive therapy in neuroblastoma and astrocytoma [225,226].

While the therapeutic synergy between phytochemicals and allopathic drugs holds considerable promise for treating neurological conditions, the associated interactions are complex and multifactorial. These effects depend on numerous factors, including dosage, compound structure, metabolic pathways, and individual variability. Therefore, it is essential to undertake more rigorous clinical evaluations, employ standardized plant extracts, and utilize advanced pharmacokinetic modeling to mitigate risks and maximize benefits. Such strategies will ensure the safe and effective integration of phytochemicals into neurological disease management protocols.

## 7. Conclusions

The intersection of phytochemicals and allopathic therapy presents a promising new frontier in the treatment of neurological diseases. Conditions such as Alzheimer’s disease, Parkinson’s disease, epilepsy, spinal injuries, and traumatic brain injuries continue to pose significant clinical challenges, often characterized by limited therapeutic efficacy and severe side effects. Although allopathic treatments provide essential symptom relief and disease management, they frequently lack the capacity for neuroprotection or regeneration.

Phytochemicals—natural compounds derived from plants, including flavonoids, alkaloids, and terpenes—have demonstrated antioxidant, anti-inflammatory, and neuroprotective properties in both experimental and clinical studies. These phytochemicals have the potential to augment pharmaceutical interventions, thereby enhancing therapeutic effects or mitigating undesirable side effects. However, the co-administration of phytochemicals may also introduce risks, such as adverse drug-drug interactions, alterations in drug metabolism, or reduced therapeutic efficacy. Therefore, a comprehensive understanding of phytochemical interactions with conventional neurologic medications is imperative.

The integration of plant-based agents into clinical therapy requires rigorous research, standardization, and meticulous consideration of their efficacy and safety. As the body of scientific evidence grows, an expanded holistic and integrative approach to neurologic therapeutics—utilizing the strengths of both phytotherapy and contemporary pharmacy—has the potential to transform therapeutic outcomes for patients and broaden the arsenal available for addressing neurodegenerative and neurotraumatic disorders.
